# Oncogenic KRAS Requires Complete Loss of BAP1 Function for Development of Murine Intrahepatic Cholangiocarcinoma

**DOI:** 10.3390/cancers13225709

**Published:** 2021-11-15

**Authors:** Rebecca Marcus, Sammy Ferri-Borgogno, Abdel Hosein, Wai Chin Foo, Bidyut Ghosh, Jun Zhao, Kimal Rajapakshe, James Brugarolas, Anirban Maitra, Sonal Gupta

**Affiliations:** 1 Department of Translational Molecular Pathology, University of Texas MD Anderson Cancer Center, Houston, TX 77030, USA; sferri@mdanderson.org (S.F.-B.); abdelhosein@gmail.com (A.H.); BGhosh@mdanderson.org (B.G.); JZhao6@mdanderson.org (J.Z.); kirajapakshe@mdanderson.org (K.R.); AMaitra@mdanderson.org (A.M.); docgupta24@gmail.com (S.G.); 2 Department of Surgical Oncology, Saint John’s Cancer Institute, Santa Monica, CA 90404, USA; 3 Department of Gynecologic Oncology and Reproductive Medicine, University of Texas MD Anderson Cancer Center, Houston, TX 77030, USA; 4 Advocate Aurora Health, Vince Lombardi Cancer Clinic, Sheboygan, WI 53081, USA; 5 Department of Pathology, University of Texas MD Anderson Cancer Center, Houston, TX 77030, USA; WFoo@mdanderson.org; 6 Kidney Cancer Program, Simmons Comprehensive Cancer Center, Department of Internal Medicine, University of Texas Southwestern Medical Center, Dallas, TX 75390, USA; james.brugarolas@utsouthwestern.edu; 7 Sheikh Ahmed Center for Pancreatic Cancer Research, University of Texas MD Anderson Cancer Center, Houston, TX 77030, USA

**Keywords:** intrahepatic cholangiocarcinoma, mouse model, BAP1, KRAS, ferroptosis, SLC7A11

## Abstract

**Simple Summary:**

Intrahepatic cholangiocarcinoma (ICC) is a primary liver cancer that currently has limited treatment options. As a result, patients with this disease generally have a poor prognosis. Previous studies have demonstrated that mutations in *KRAS* and loss of *BRCA-1-associated protein 1* (*BAP1*) are frequently found in ICC. We developed a mouse model for ICC that incorporates *KRAS* and *BAP1* mutations in a liver-dependent fashion to aid in the improvement of our understanding of this devastating disease. Our findings suggest that complete loss of BAP1 function combined with mutant KRAS appears to be a requirement for inducing ICC formation within the liver.

**Abstract:**

Intrahepatic cholangiocarcinoma (ICC) is a primary biliary malignancy that harbors a dismal prognosis. Oncogenic mutations of *KRAS* and loss-of-function mutations of *BRCA1-associated protein 1* (*BAP1*) have been identified as recurrent somatic alterations in ICC. However, an autochthonous genetically engineered mouse model of ICC that genocopies the co-occurrence of these mutations has never been developed. By crossing *Albumin*-Cre mice bearing conditional alleles of mutant *Kras* and/or floxed *Bap1,* Cre-mediated recombination within the liver was induced. Mice with hepatic expression of mutant *Kras*^G12D^ alone (KA), bi-allelic loss of hepatic *Bap1* (B^homo^A), and heterozygous loss of *Bap1* in conjunction with mutant *Kras*^G12D^ expression (B^het^KA) developed primary hepatocellular carcinoma (HCC), but no discernible ICC. In contrast, mice with homozygous loss of *Bap1* in conjunction with mutant *Kras*^G12D^ expression (B^homo^KA) developed discrete foci of HCC and ICC. Further, the median survival of B^homo^KA mice was significantly shorter at 24 weeks when compared to the median survival of ≥40 weeks in B^het^KA mice and approximately 50 weeks in B^homo^A and KA mice (*p* < 0.001). Microarray analysis performed on liver tissue from KA and B^homo^KA mice identified differentially expressed genes in the setting of BAP1 loss and suggests that deregulation of ferroptosis might be one mechanism by which loss of BAP1 cooperates with oncogenic Ras in hepato-biliary carcinogenesis. Our autochthonous model provides an in vivo platform to further study this lethal class of neoplasm.

## 1. Introduction

Cholangiocarcinomas are a group of primary liver cancers arising from the biliary epithelium. These aggressive cancers are the second most common primary hepatic malignancy after hepatocellular carcinoma (HCC), and they currently represent 15% of all primary liver tumors and 3% of gastrointestinal cancers [1]. Traditionally, cholangiocarcinomas are classified based on anatomic site of origin and include carcinomas arising from the intrahepatic, perihilar, and extrahepatic biliary tree [2]. While the incidence of perihilar and extrahepatic disease has been decreasing [3,4,5], the incidence and mortality related to intrahepatic cholangiocarcinoma (ICC) have been rising over the past several decades in many parts of the world [1,5]. This is perhaps most notable within the Western Hemisphere [6,7], as there has been a doubling of the annual incidence of reported ICC in the United States since the 1970s [8]. This trend was noted to have accelerated during the last decade, with an annual percentage increase of >4% [8].

At present, the only potential curative treatment for ICC is surgical resection; however, the majority of patients have advanced disease at the time of diagnosis [9], and ultimately only 15% of patients are eligible for curative resection [10,11]. Multiple factors contribute to the typically late presentation of ICC, including that this disease process is often asymptomatic, as opposed to perihilar and extrahepatic cholangiocarcinoma, which present earlier due to the development of biliary and/or pancreatic duct obstruction [11,12]. Moreover, even once symptoms from ICC develop, they tend to be non-specific, and diagnosis may be delayed by initial efforts to rule out more common pathologies [11,12]. ICC also lacks a unique serum biomarker to be used for disease screening and/or surveillance [11]. Finally, and even as imaging technology continues to improve, there remain limitations to the detection of and differentiation between various types of liver lesions [11,12,13]. As such, up to one-third of ICC patients thought to be eligible for curative-intent resection are found to be non-resectable due to locally advanced or metastatic disease at the time of surgery [11,13,14].

Among those patients who are able to undergo curative-intent resection, treatment failure is common, with recurrence in as many as 66% of patients and median overall survival of only 28–36 months [10,15]. Unfortunately, and despite the advances made in available systemic and targeted therapies for many malignancies, the available treatment options for patients with ICC remain limited [11,16,17,18,19,20]. Moreover, best practice recommendations continue to be debated [21] with respect to which patients should receive systemic therapy, the therapeutic regimen they should receive, the timing of systemic therapy in relation to surgical intervention, and the use of associated radiotherapy [1,9,13,22,23,24,25]. This translates into the poor prognosis associated with ICC, with <5% of patients being alive 5 years after diagnosis [10,26,27].

Despite its dismal prognosis and rising incidence, there are still substantial deficits in our current understanding of ICC, including the mechanisms of biliary carcinogenesis. This is at least in part due to an incomplete assortment of experimental tools for studying ICC, including a paucity of available cell lines and faithful genetically engineered mouse models (GEMMs). Recent molecular studies of ICC tumors have identified recurrent somatic mutations in several oncogenes and tumor suppressor genes. For example, oncogenic *KRAS* mutations have been observed in 5–27% of ICCs [28,29]. This is not unexpected given that Ras signaling has been demonstrated to be deregulated in numerous human tumors and that *KRAS* is the most commonly mutated oncogene in human cancers [29,30,31]. Other recurrently mutated oncogenes that have been identified in ICC include *IDH1/2*, *BRAF*, and *PIK3CA* [28].

Recurrent loss-of-function mutations of the *breast cancer type 1 susceptibility protein* (*BRCA1*)*-associated protein 1* (*BAP1*) have also been identified in ICC, along with such mutations in *TP53*, *ARID1A/B*, *PBRM1*, *STK11*, *PTEN*, and *CKDN2A* [28]. Specifically, *BAP1* has been found to be mutated in 7–32% of Western ICCs [28]. *BAP1* mutations are also seen in Eastern ICCs, which are frequently associated with liver-fluke infections, albeit at slightly lower frequencies than seen among Western ICCs [29]. Previous studies have suggested that BAP1 functions as a tumor suppressor [32,33], and both germline and somatic mutations of *BAP1* have been identified in numerous tumor types, including melanoma, mesothelioma, renal cell carcinoma, and breast cancer [34,35,36,37]. BAP1 is a nuclear deubiquitinating enzyme in the ubiquitin carboxyterminal-hydrolase subfamily that is involved in chromatin remodeling [32]. Loss of BAP1 links deregulation of the cell death mechanism known as ferroptosis to carcinogenesis. Indeed, recent studies have demonstrated that BAP1 represses expression of the cystine transporter SLC7A11 and, as a consequence, inhibits cystine uptake, leading to elevated lipid peroxidation and ferroptosis-mediated cell death. Cancer-associated *BAP1* mutants lose their abilities to repress SLC7A11, leading to attenuation of ferroptosis and tumor promotion [38,39].

In this study, we sought to investigate the cooperation between Ras and BAP1 in ICC pathogenesis by generating autochthonous mice with Cre-mediated conditional activation of mutant *Kras* and/or deletion of *Bap1* alleles within the albumin (*Alb*) expressing domain, which is comprised of liver progenitor cells, as well as adult hepatocytes and cholangiocytes [40,41]. Our findings demonstrate that complete abrogation of BAP1 function might be one of the requirements for developing an ICC phenotype in mice expressing oncogenic Kras in the Alb-expressing domain, underscoring the importance of this tumor suppressor gene in ICC pathogenesis. Further, upregulation of the cystine transporter xCT (encoded by *SLC7A11*) in B^homo^KA compared to KA neoplasms suggests a potential role for ferroptosis deregulation in the tumor-promoting phenotype induced by *BAP1* loss. This work establishes a relevant autochthonous model of ICC that genocopies the co-occurrence of two recurrent mutations observed in a subset of human ICC and provides an opportunity to evaluate putative actionable pathways against this lethal disease in an in vivo setting.

## 2. Materials and Methods

### 2.1. Generation of Conditional BAP1 Mice

BAP1^L/L^ mice were kindly provided by Dr. James Brugarolas (University of Texas Southwestern Medical Center) and are now available commercially (Stock No: 031565. Jackson Laboratories, Bar Harbor, ME, USA) [42,43]. *Albumin*-Cre mice have been previously described [40,41] and were purchased from Jackson Laboratories (stock no: 016832). Lox-STOP-Lox-*Kras^G12D^* mice have also been described before [44] and were purchased from Jackson Laboratories (stock no: 019104). BAP1^L/L^ mice were crossed with Alb-Cre or Albumin-Cre; LSL-Kras^G12D^ mice to generate Albumin-Cre; BAP1^L/+^ (B^het^A), Albumin-Cre; BAP1^L/L^ (B^homo^A), Albumin-Cre; Kras^G12D^; BAP1^L/+^ (B^het^KA), and Albumin-Cre, Kras^G12D^; BAP1^L/L^ (B^homo^KA) mice. All mice were housed in a pathogen-free barrier facility with food and water *ad libitum*. Animal studies were conducted in compliance with Institutional Animal Care and Use Committee (IACUC) guidelines of the University of Texas MD Anderson Cancer Center and performed in accordance with the NIH guidelines (https://grants.nih.gov/grants/olaw/guide-for-the-care-and-use-of-laboratory-animals.pdf) (Accessed on 15 July 2015) for use and care of live animals under the protocol number 00001937-RN00 (expiring May 2022). PCR was performed to confirm the genotype of mice using DNA obtained from tails.

### 2.2. Genotyping PCR

DNA extraction from mouse tail clips obtained at 7–10 days of age was performed using REDExtract-N-Amp™ Tissue PCR Kit (Cat#R4775, Sigma-Aldrich, St. Louis, MO, USA) following the manufacturer’s protocol. In brief, tail clips were combined with 100 μL of extraction solution and 25 μL of tissue preparation solution and mixed by vortexing. Samples were incubated for 15 min at room temperature followed by incubation at 95 °C for 5 min. A total of 100 μL of neutralization solution was added to each sample and again mixed by vortexing.

The extracted DNA was subsequently used to perform genotyping PCR. The primers of interest were combined with the extracted DNA, double-distilled water, and REDExtract-N-Amp™ PCR Ready Mix (Cat#R4775, Sigma-Aldrich, St. Louis, MO, USA) to obtain a total volume of 20 μL per reaction. PCR was performed using an Applied Biosystems Thermal Cycler; individual thermal protocols were primer-specific (Supplementary Table S1). A total of 2.5–8 μL of the final samples were run out on 1.5% agarose gels for BAP1 and Kras specimens and 3% agarose gels for Alb-Cre specimens. Primer sequences and expected amplicon sizes are found in Supplementary Figure S1.

### 2.3. Histology and Immunohistochemistry

Mouse tissue obtained during necropsies was fixed in 10% neutral buffered formalin for 72 h. They were subsequently processed, embedded in paraffin, sectioned, and stained with hematoxylin and eosin (H&E), and reticulin. For immunohistochemistry, 5 μm sections were incubated with primary antibodies as described previously [45]. For analysis of marker expression, at least three mice per genotype were characterized, and ImageJ software was used for the quantification of tissue sections. Antibodies used are as follows: Anti-BAP1 (Cat#PA5-12061, Thermo Fisher Scientific, Waltham, MA, USA), anti-CK-19 (Cat#MABT913, Millipore Sigma), anti-HepPar1 (Cat#NBP2-45272, Novus Biologicals), and anti-SLC7A11 (Cat#NB300-218, Novus Biologicals). ImmPRESS-HRP-linked anti-rat (Cat#MP744415) was purchased from Thermo Fisher Scientific and Mouse on Mouse ImmPRESS-HRP-linked anti-mouse (Cat#MPX-2402-15) from Maravai LifeSciences.

### 2.4. Microarray Analysis

After histopathologic confirmation of the presence of primary liver tumors, total RNA was extracted from three KA and three B^homo^KA flash-frozen tumors using the RNeasy Mini kit (Cat#74106, Qiagen) according to the manufacturer’s instructions. Samples were submitted to the Advanced Technology Genomics Core (ATGC) facility at the University of Texas MD Anderson Cancer Center. Briefly, the concentration of total RNA was assessed using the Nanodrop ND-1000 Spectrophotometer (Thermo Fisher Scientific, Waltham, MA, USA). Once the sample concentration was determined, the integrity of the total RNA was assessed using the Agilent 2100 Bioanalyzer Nanochip assay. Samples with a minimum concentration of 33 ng/ul were selected for target amplification with the Whole Transcript (WT) Plus assay (Affymetrix). A total of 100 ng of total RNA was used to process the samples for whole transcriptome expression analysis with the WT Plus assay.

Samples were reversed transcribed to generate amplified, fragmented, and biotinylated sense-strand cDNA (sscDNA), according to the manufacturer’s standard protocol. A total of 5.2 µg of fragmented and labeled sscDNA was then hybridized to Affymetrix Mouse Transcriptome Array 1.0 ST at 45 °C for 16 h and subsequently washed and stained using Affymetrix proprietary reagents in the GeneChip Fluidics Station 450 and scanned in the GeneChip Scanner 3000 7G (Affymetrix).

CEL files generated after Mouse Transcriptome 1.0 ST GeneChip scanning were uploaded onto the Expression Console software. The CEL files were pre-processed and normalized using the robust multichip average (RMA) algorithm implemented in oligo [46] package within R statistical software (R, v4.0.2). Differentially expressed genes between the experimental groups were identified using the Bioconductor limma [47] package imposing a filtering criterion of fold change >2 (<0.5) and *p* < 0.05. Clustering and heatmaps were generated using Matplotlib, NumPy, and SciPy libraries under Python. Gene Set Enrichment Analysis (GSEA) using Molecular Signature Database (MSigDB) [48] was performed to find enriched pathways (*q* < 0.25).

### 2.5. Statistical Analysis

Statistical analyses were performed using Prism 8 (GraphPad, San Diego, CA, USA). Statistical significance was determined using the unpaired Student’s *t*-test with Welch’s correction and two-way ANOVA with Sidak’s post-hoc test, as appropriate. For all experiments with error bars, standard deviation (SD) was calculated to indicate the variation within each experiment and data, and values represent mean ±SD. Kaplan–Meier method and log-rank test were used for survival analysis of mice. A *p*-value of < 0.05 was regarded as statistically significant.

## 3. Results

**Figure 1:**
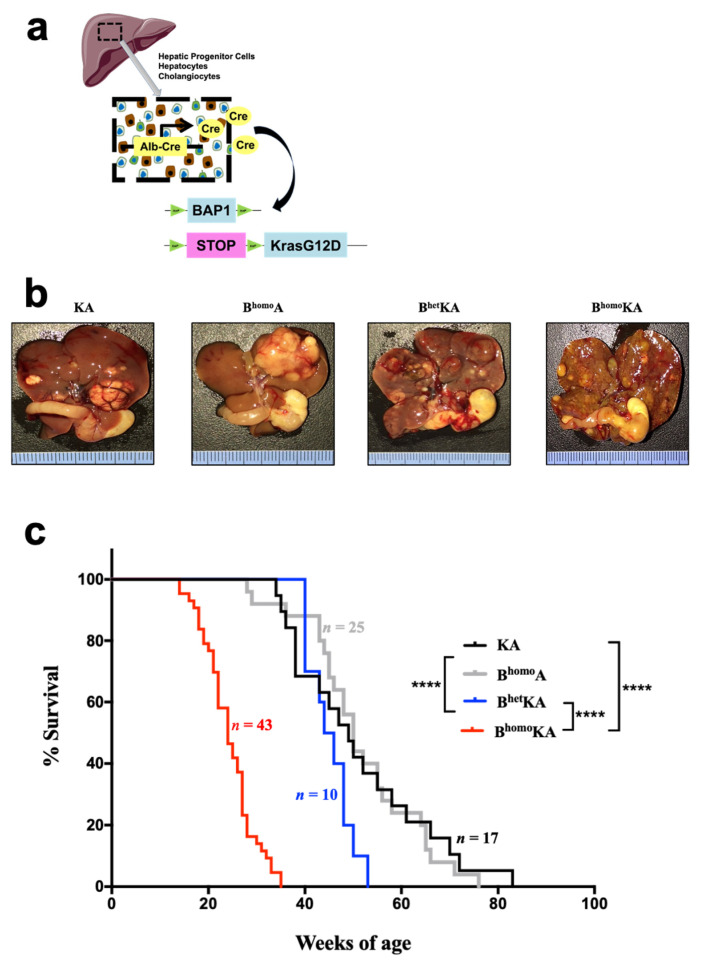
Mice with constitutional KrasG12D activation and BAP1 deletion in the hepatic epithelium develop primary liver tumors. (**a**) Modeling strategy to generate compound mutant mice. Mice harboring *Albumin*-Cre transgene, Lox-STOP-Lox-*Kras*^G12D^, and *BAP1*^L/L^ were created to conditionally activate *Kras*^G12D^ and delete BAP1 in the hepatic epithelium; (**b**) in situ gross tumor nodules throughout the hepatic parenchyma with associated hepatomegaly in experimental cohorts; (**c**) Kaplan–Meier survival analysis for KA (*n* = 17), B^homo^A (*n* = 25), B^het^KA (*n* = 10), and B^homo^KA (*n* = 43) cohorts. Two-tailed unpaired Student’s t-test was used for data analysis and considered significant if ****, *p* < 0.0001.

We generated a genetically engineered mouse model (GEMM) with a conditional knockout allele for *Bap1* (BAP1^L/L^) and a conditionally activated allele for *Kras* (LSL-*Kras*^G12D^) (Figure 1a). In our GEMM cohorts, Cre expression was under the control of the *Albumin* promoter, resulting in expression of oncogenic *Kras* and loss of *Bap1* in liver progenitor cells during late embryogenesis, as well as within Alb-expressing hepatocytes and cholangiocytes of adult mice [41,49]. Of note, several previously described ICC GEMMs have used the *Albumin* promoter to target genetic alterations of interest to the liver [40,50,51,52]. The *Bap1*^L/L^ allele sustains Cre-mediated excision of exons 4 and 5, resulting in a functionally inactive gene as previously described [53] (Figure 1a). The *LSL-Kras*^G12D^ allele results in oncogenic *Kras* expression at endogenous levels following Cre-mediated excision of a transcriptional stop element [30]. Compound mutant mice with the following genotypes were achieved through multiple generations of crossbreeding: Alb-Cre; *Kras*^G12D^ (KA), Alb-Cre; *BAP1*^L/L^ (B^homo^A), Alb-Cre; *Kras*^G12D^; *BAP1*^L/+^ (B^het^KA), and Alb-Cre; *Kras*^G12D^; BAP^L/L^ (B^homo^KA) (Figure 1a).

### 3.1. Tissue-Specific Concomitant Expression of Oncogenic Kras and Bap1 Loss Results in Primary Liver Tumors and Decreased Survival

We first evaluated the gross intraabdominal findings and survival of all experimental cohorts. KA, B^homo^A, B^het^KA, and B^homo^KA mice were produced in the expected Mendelian frequencies. No cohort demonstrated evidence of early developmental abnormalities. Animals were monitored via serial examinations until they developed signs of illness, including abdominal distension and bloating, cachexia, jaundice, diminished activity, and anorexia. Mice demonstrating one or more of these moribund criteria were euthanized, with the exception of those mice included for timed necropsies.

At the time of necropsy, all moribund animals were found to have hepatomegaly and solid liver tumors of various sizes located throughout the liver parenchyma (Figure 1b). These macroscopic findings corresponded to the abdominal distension observed on serial animal exams. Hepatic tumors presented as isolated nodules or, more often, as multiple independent lesions. Rarely, fluid-filled cystic lesions were found in addition to solid tumors. Some uncommon features observed at necropsy (all in less than 10% of mice) were areas of cystic degeneration or local invasion into adjacent organs (e.g., stomach, duodenum, pancreas, and spleen).

The majority of KA mice lived for several months before exhibiting any signs of disease, after which they survived several additional weeks before requiring euthanasia for the severity of symptoms (Figure 1c). The median survival of this cohort was 49 weeks (Figure 1c). The natural history of disease in the B^homo^A mice was comparable, with no significant differences in disease penetrance, timing of symptom onset, or rate of disease progression as compared to KA mice, with a median survival of 50 weeks (Figure 1c). The heterozygous loss of *Bap1* allele on a backdrop of oncogenic *Kras* expression in the B^het^KA mice resulted in acceleration of disease onset, with a median survival of 45 weeks, while the B^homo^KA mice demonstrated the most aggressive natural history among all genotypes, with a median survival of only 24 weeks (*p* < 0.0001 for B^homo^KA versus other cohorts) (Figure 1c). Taken together, these data demonstrate that concomitant oncogenic *Kras* and homozygous deletion of *Bap1* results in reduced survival compared to the presence of either of these mutations individually or heterozygous deletion of *Bap1*.

### 3.2. Kras^G12D^ Activation in Combination with Homozygous Bap1 Deletion Results in Development of ICC

**Figure 2:**
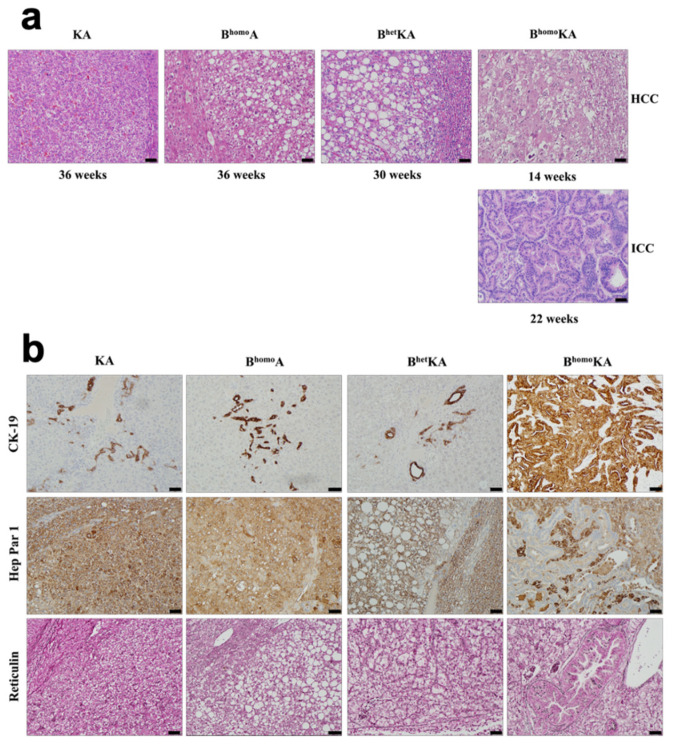
GEMM experimental cohorts have distinct histologic phenotypes. (**a**) KA, B^homo^A, and B^het^KA mice exhibit HCC only by H&E, while B^homo^KA mice have both ICC and HCC lesions; (**b**) CK-19, Hep Par 1, and reticulin staining demonstrates HCC lesions in all cohorts and both HCC and ICC in B^homo^KA mice. CK19 highlights normal ducts in KA, B^homo^A, and B^het^KA mice, while ICC foci are strongly positive in B^homo^KA mice. Conversely, Hep Par 1 stains positive among HCC foci in KA, B^homo^A, and B^het^KA mice, as well as in HCC foci of B^homo^KA mice; however, it is negative in ICC foci of the latter. Reticulin highlights abnormal architecture in the HCC of all four mouse genotypes. Images were taken with 200× magnification. Scale bar is 50 µm.

We next performed histopathologic analysis of the livers from each experimental cohort. Mice from all cohorts eventually developed hepatocellular carcinoma (HCC), albeit at different ages and with variable penetrance (Figure 2a, Table 1, Supplementary Figure S2). To confirm the effectiveness of *Bap1* gene deletion in B^homo^A, B^het^KA, and B^homo^KA mice, we evaluated BAP1 protein expression via immunohistochemistry (IHC) (Supplementary Figure S3). As expected, the B^homo^A and B^homo^KA mice show no BAP1 expression by IHC, while B^het^KA show decreased BAP1 expression as compared to KA control mice (Supplementary Figure S3). Of note, BAP1 expression is lost within both the normal hepatic parenchyma (Supplementary Figure S3a) and all primary liver lesions (i.e., ICC and HCC) (Supplementary Figure S3b).

In contrast to the other genotypes, microscopic analyses of the liver in B^homo^KA mice revealed a mixed phenotype consisting of both ICC and HCC (Figure 2a). At 4–8 weeks of age, all experimental cohorts had normal liver histology (Supplementary Figure S2a–c) with the exception of B^homo^KA mice, in which bile duct hyperplasia was observed (Supplementary Figure S2d). Abnormal liver findings, including the fatty transformation of hepatocytes, steatohepatitis, and parenchymal congestion, were eventually observed in all mice. Specifically, KA mice developed these changes between 20 and 28 weeks, B^homo^A mice between 24 and 32 weeks, B^het^KA mice between 20 and 24 weeks, and B^homo^KA mice developed these changes the earliest between 4 and 8 weeks of age (Supplementary Figure S2). The spectrum of hepatocellular disease included a variety of lesions, from hepatic adenomas to well-differentiated HCC to, in some cases, poorly differentiated HCC. The timing of development varied between experimental cohorts, with these pathologic findings occurring in B^homo^KA mice as early as 12 weeks of age and in KA mice as late as 40 weeks (Supplementary Figure S2). Furthermore, the proportion of mice within each cohort that developed HCC was lower in the B^homo^A mice (60%) mice than in the other cohorts (87–94%, Table 1). The histologic spectrum of liver disease observed was similar across multiple generations of each experimental cohort.

Notably, histological findings of ICC were only observed in the B^homo^KA mice (Table 1). Specifically, microscopic changes consistent with bile duct dysplasia developed by 20 weeks of age, and foci of frank ICC were observed within 20–24 weeks (Supplementary Figure S2d). The degree of ICC differentiation varied between lesions, and even within the same lesion, but typically featured poorer differentiation in older mice. Overall, the timed necropsies show the development of ICC only in mice with concomitant *Kras* activation and homozygous *Bap1* deletion. Furthermore, the earlier death exhibited in the B^homo^KA mice may be, at least in part, due to the development of the ICC.

To confirm the presence of two distinct differentiation lineages of neoplasm within our experimental cohorts, we stained tissue sections for markers of the biliary epithelium (cytokeratin 19; CK-19) and hepatocytes (hepatocyte-specific antigen 1; Hep Par 1) that have previously been used to distinguish ICC from HCC clinically [54,55,56,57,58,59]. Predictably, IHC analysis with CK-19 demonstrated robust staining of bile duct hyperplasia, dysplasia, and ICC in B^homo^KA mice, while only normal-appearing bile ducts stained positively with CK-19 in all other experimental cohorts (Figure 2b). Moreover, IHC for Hep Par 1 showed expression in hepatic adenomas and frank HCC in all experimental cohorts, while biliary epithelium-derived lesions did not show any staining (Figure 2b). Finally, staining with reticulin demonstrated a loss of the normal hepatic architecture in areas of hepatic adenomas and HCC development, but intact architecture within normal bile ducts, biliary hyperplasia, and dysplasia, and frank ICC (Figure 2b). This is reminiscent of patterns seen in corresponding human disease processes where architectural disruption occurs with the development of hepatic adenomas and HCC, as well as other hepatocellular proliferative diseases, but not with ICC [60,61,62].

It should be noted that primary lung adenocarcinomas were observed in subsets of both KA and B^het^KA mice (Supplementary Figure S4). Specifically, 60% of KA and 55% of B^het^KA mice ≥24 weeks of age demonstrated lung lesions. These lesions were not seen in B^homo^KA mice, likely due to the accelerated natural history of the liver pathology in this mouse cohort. We confirmed by histology and CK-19/Hep Par 1 IHC that the pulmonary lesions were primary lung adenocarcinomas, not metastatic HCC (Supplementary Figure S4). Notably, low levels of albumin expression exist in the alveolar cells of adult lungs per the Human Protein Atlas and are also well reported in fetal lung tissues [63]. Therefore, the observed lung lesions likely represent breakthrough Cre expression in this organ.

### 3.3. Transcriptomic Profiling of B^homo^KA Mouse Tumors Identifies Potential Effector Pathways of Tumor Promotion Caused by Loss of BAP1

**Figure 3:**
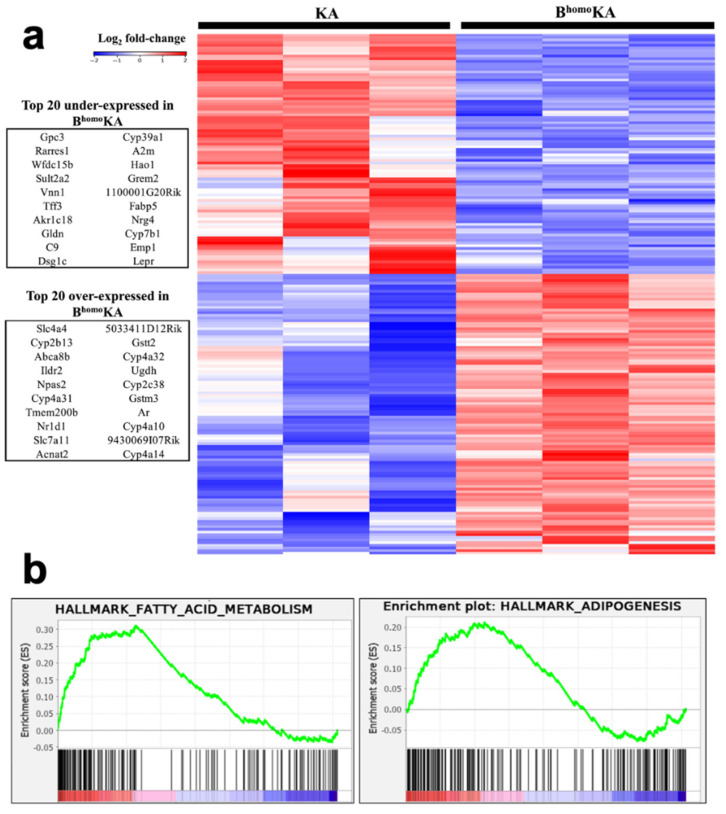
Hepatic tumors from KA and B^homo^KA experimental GEMM cohorts have unique gene expression patterns. (**a**) KA and B^homo^KA liver lesions have differential gene expression by Affymetrix microarray. Supervised clustering heatmap of Affymetrix microarray data (3 KA samples versus 3 B^homo^KA samples, log_2_ fold change >2, *p* < 0.05). The top 20 under- and overexpressed genes in the B^homo^KA cohort are pictured to the left of the heatmap; (**b**) Gene set enrichment analysis (GSEA) of differentially expressed transcripts in microarray of KA and B^homo^KA hepatic tumors show positive enrichment in hallmark gene signatures for fatty acid metabolism and adipogenesis.

To further characterize the role of *Bap1* as a tumor suppressor gene, transcriptomic analysis was performed on harvested liver tumors from KA and B^homo^KA mice, with three mice per genotype used for microarray analysis. This analysis identified multiple differentially expressed genes (DEGs) (>2-fold change in either direction, *p* < 0.05) (Figure 3), a subset of which were further validated (see below). In addition, Gene Set Enrichment Analysis (GSEA) of DEGs nominated cellular pathways that were significantly enriched in the B^homo^KA compared to KA mice, such as enrichment of fatty acid metabolism and adipogenesis signatures, suggesting altered lipid metabolism as a potential driver of accelerated tumorigenesis observed in the setting of bi-allelic *BAP1* deletions (Figure 3). We confirmed these results in the human setting by using the TCGA-PAAD data set. Specifically, a low *BAP1* expression cohort was compared to a high *BAP1* expression cohort, and GSEA was performed. Similar to our findings in B^homo^KA mice, fatty acid metabolism and adipogenesis pathways were found to be enriched within the low *BAP1* expression cohort.

**Figure 4:**
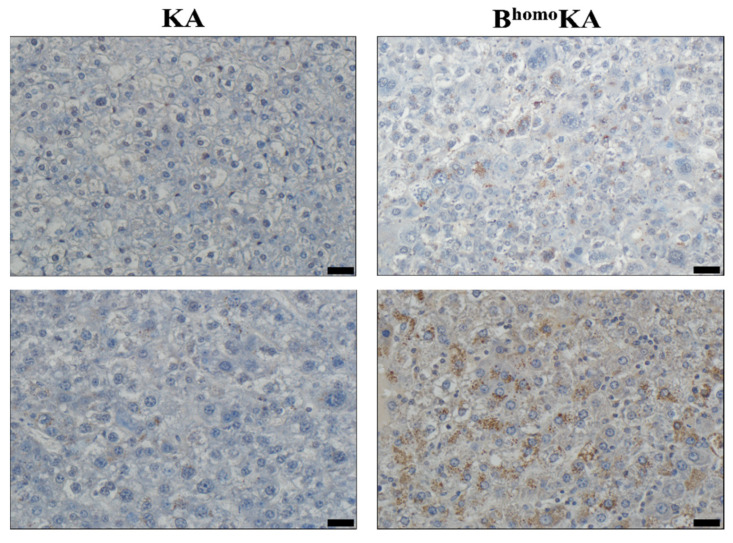
Loss of BAP1 is associated with increased hepatic expression of the cystine/glutamate transporter (SLC7A11/xCT) in GEMM experimental cohorts. Immunohistochemical (IHC) staining for SLC7A11/xCT shows strong staining throughout the neoplastic tissue of B^homo^KA mice as compared to KA, which does not demonstrate staining. Images in the upper panel were taken at 200× magnification. Images in the lower panel were taken at 400× magnification, which more clearly demonstrates the strong granular SLC7A11/xCT cytoplasmic staining in neoplastic cells of B^homo^KA liver sections. Scale bar is 50 µm for 200× images and 25 µm for 400× images.

*SLC7A11* was found to be one of the highest overexpressed genes in B^homo^KA tumors compared to KA tumors (Figure 3). Recent studies have highlighted the potential role of altered cystine transport as the mechanism by which loss of BAP1 promotes tumorigenesis, specifically via upregulation of the cystine/glutamate antiporter xCT that is encoded by *SLC7A11* [36]. Therefore, IHC for SLC7A11/xCT expression was performed on harvested liver sections to validate the microarray findings. While KA tumors had minimal to absent SLC7A11/xCT expression within neoplastic tissues, robust SLC7A11/xCT expression was observed in all of the examined B^homo^KA tumors, particularly so within the HCC foci (Figure 4). Surprisingly, in addition to faint membranous staining, we noted strong granular SLC7A11/xCT staining in the cytoplasm of neoplastic cells of B^homo^KA mice (Figure 4), the significance of which is unclear. Nonetheless, the IHC data confirmed the microarray findings that SLC7A11/xCT was upregulated within B^homo^KA compared to KA tumors, validating prior data that loss of BAP1 upregulates this cystine transporter and regulator of ferroptosis in neoplastic cells [36,37].

## 4. Discussion

In this study, we have established a novel autochthonous mouse model of ICC that incorporates bi-allelic *Bap1* deletion in conjunction with expression of mutant *Kras* within the *Albumin*-expressing progenitor population in the developing liver. Our data establish that bi-allelic *Bap1* deletion is a requirement to elicit an ICC phenotype in the presence of mutant *Kras*, as observed in B^homo^KA animals. Mice that retain even partial Bap1 function (B^het^KA mice) demonstrate pure HCC without ICC foci, reiterating the importance of this complete loss-of-function requirement. These findings are in line with previous studies that suggest *BAP1* functions as a tumor suppressor, and, as such, *BAP1*-induced pathogenesis should follow Knudson’s two-hit hypothesis [32,33].

There have been previous reports of ICC GEMMs, most of which, like our B^homo^KA model, develop combined HCC and ICC. These include models with liver-specific *Pten* and *Smad4* deletion [50], *Kras* activation and *TP53* deletion [40], *Kras* activation and *Pten* deletion [64], *Sav1* deletion [65], and expression of the Notch receptor intracellular domain (*NICD*) [66]. For example, O’Dell and colleagues demonstrated that mutant *Kras* expression alone led to ICC development with low penetrance and after a long latency, while the combination of mutant *Kras* expression and *TP53* deletion resulted in near-complete penetrance of ICC development, often with evidence of metastatic disease, in addition to HCC development [40]. More recently, a mouse model incorporating liver-specific mutant *Kras* expression and *Pten* deletion demonstrated mixed ICC and HCC when heterozygous *Pten* deletion was present, but pure ICC development with *Kras* activation and homozygous *Pten* deletion [64].

Recent molecular studies of human tumor samples have demonstrated that somatic mutations in *BAP1* and *KRAS* are among the most frequent mutations present in ICC [28]. To the best of our knowledge, our model represents the first murine model of ICC to incorporate a *Bap1* deletion. The tumors induced in the B^homo^KA model recapitulate many of the key histologic features of human ICC, including demonstration of the multistep progression of histopathological changes from bile duct hyperplasia to dysplasia, carcinoma in situ, and culminating in ICC. Both well-differentiated ICC, characterized by intact glandular architecture and mucin production, and poorly differentiated ICC with minimal gland formation, large pleomorphic cells, nuclear atypia, and frequent mitoses were seen.

In our model, liver-specific deletion of *Bap1* and *Kras* activation were regulated by Cre recombinase expression driven by the *Albumin* promoter, resulting in Cre-mediated recombination in liver progenitor cells in late embryonic development, as well as in mature hepatocytes and cholangiocytes. Based on morphological and immunohistochemical studies, HCC and ICC were considered to originate from mature hepatic parenchymal cells, hepatocytes, and cholangiocytes, respectively [67]. Over time, evidence has accumulated that suggests multiple potential cells of origin of both HCC and ICC. These range from more mature hepatocytes and cholangiocytes to less-differentiated oval cells and hepatoblasts and, finally, to the least-differentiated liver stem cells [68,69,70]. As our model targets liver-specific *Bap1* deletion and oncogenic *Kras* expression to multiple Alb-expressing cell types (an inherent pitfall of the *Alb*-Cre model), the precise cell or cells of origin in our GEMMs remains speculative in the absence of lineage tracing studies. These represent a future direction of our work, as lineage tracing studies will help to clarify the cell(s) of origin for the ICC found in our model and add to the current body of literature that exists regarding potential cells of origin for both ICC and HCC.

Our finding that ICC only developed in mice with homozygous deletion of *Bap1* and *Kras* activation suggests that complete loss of *Bap1* cooperates with oncogenic *Kras* to either preferentially drive the putative cell(s) of origin down the pathway of cholangiocyte differentiation and/or transdifferentiation of hepatocytes into biliary-like cells. As mentioned, this finding is aligned with the fact that *BAP1* is thought to function as a tumor suppressor and follow a two-hit model to result in a phenotypic change. Future studies, including microarray and GSEA analyses on tissue obtained from B^het^KA mice, may help to support this hypothesis.

The fact that ICC development was not observed in B^homo^A mice suggests that loss of *BAP1* alone does not alter the molecular underpinnings of the cell(s) of origin substantially enough to enable malignant transformation, and we hypothesize that only via the coupling of *BAP1* loss with constitutional activation of an oncogene are cells able to undergo malignant transformation. This is consistent with many existing ICC GEMMs that, as previously described, combine loss of a tumor suppressor and gain of an oncogenic driver to induce ICC development. Alternatively, ICC may only be observed in B^homo^KA mice because heterozygous loss of *BAP1* and/or homozygous loss of *BAP1* without *KRAS* activation results in ICC development with low penetrance or after a long latency. As such, and due to the more rapid development of other primary liver disease (i.e., HCC) and off-target effects (e.g., primary lung adenocarcinoma) in B^het^KA and KA mice, it is possible these mice succumbed to other disease processes before ICC development occurred.

Ferroptosis is a metabolic, stress-induced, non-apoptotic form of regulated cell death [38]. The role of ferroptosis in oncogenesis is a topic of ongoing investigation. Recently, BAP1 was found to link ferroptosis to tumor suppression [38]. Specifically, it was found that *Bap1* functions as a tumor suppressor gene by inhibiting cystine uptake into cells, thereby rendering them more sensitive to ferroptosis. It does so by repressing the expression of xCT via deubiquitination of H2Aub on the *SLC7A11* promoter, which encodes for this protein. xCT is the catalytic subunit of the cystine/glutamate antiporter, the major transporter of extracellular cystine. Our microarray analysis revealed that *SLC7A11* was overexpressed in B^homo^KA tumors as compared to KA tumors. Furthermore, IHC validated increased levels of the xCT protein within the neoplastic lesions found in B^homo^KA livers, compared to minimal expression in KA mice. These results suggest that in B^homo^KA mice, the lack of *SLC7A11* repression by BAP1 might enable malignant cholangiocytes to evade ferroptosis and thereby undergo malignant transformation. This hypothesis is further supported by our findings on GSEA analysis of DEGs between B^homo^KA and KA mice, which demonstrate enrichment of apoptosis pathways in the latter mouse cohort.

## 5. Conclusions

We describe a novel GEMM for ICC that is driven by liver-specific bi-allelic *Bap1* deletion and expression of oncogenic *Kras*. Our autochthonous model demonstrates that complete loss of *Bap1* function is a requirement for developing an ICC phenotype in the setting of mutant *Kras* expression. This GEMM represents a new tool in the armamentarium for studying ICC, especially that of Western origin, and improving our understanding of the disease process. Continued investigations using our mouse model should provide a better understanding of the pathogenesis of ICC (e.g., markers associated with early changes during ICC initiation that can be used for screening and enable earlier disease detection; mechanisms underlying tumor progression) and facilitate the development and preclinical testing of new therapies and/or chemoprevention for an increasingly prevalent and persistently fatal disease. Finally, as our GEMM results in the highly reproducible development of ICC, it provides a foundation for testing the influence of additional genetic lesions on ICC tumor biology, which may enable the development of therapies that target specific genomic alterations and/or molecular phenotypes.

## Figures and Tables

**Table 1:** Hepatic genotypic and phenotypic differences between GEMM experimental cohorts. Total *n* includes necropsies performed as part of both survival analyses and timed necropsy analyses after the median survival of each cohort was reached.

Genotype	Mean Survival, Range (wks)	Hepatic Histopathologic Findings	Mice With HCC, *n* (%)	Mice With ICC, *n* (%)
KA	51, 34–83	Fatty metamorphosis of hepatocytes, steatosis, parenchymal congestion, adenomas, HCC	13 (87)	0 (0)
B^homo^A	52, 28–76	Fatty metamorphosis of hepatocytes, parenchymal congestion, adenomas, HCC	15 (60)	0 (0)
B^het^KA	45, 40–53	Dilated blood vessels, adenomas, HCC	16 (94)	0 (0)
B^homo^KA	24, 14–35	Fatty metamorphosis of hepatocytes, steatosis, steatohepatitis, parenchymal congestion, biliary hyperplasia, adenomas, HCC, ICC	33 (87)	38 (100)

## Data Availability

The data presented in this study are deposited in NCBI’s Gene Expression Omnibus under GEO Series accession number GSE183554 (https://www.ncbi.nlm.nih.gov/geo/query/acc.cgi?acc=GSE183554). To review GEO accession GSE183554: Go to https://urldefense.com/v3/__https://www.ncbi.nlm.nih.gov/geo/query/acc.cgi?acc=GSE183554__;!!PfbeBCCAmug!yxRfs7maVWvBKsufS2dLRYJ49lon75ndjapD679Ra3fpYnvFB_QvHvgKmgX6AkOQL3vNCQ$. Enter token snelucggljgjtcr into the box. (Accessed on 21 September 2021).

## Supplementary Materials

The following are available online at https://www.mdpi.com/article/10.3390/cancers13225709/s1, Table S1: Genotyping thermal protocols, Figure S1: Mouse genotyping protocol, Figure S2: Hepatic histopathologic time-lapse of GEMM experimental cohorts, Figure S3: Validation of BAP1 protein expression loss, Figure S4: GEMM experimental cohorts with constitutional KRAS activation develop lung lesions.
